# Les valvulopathies cardiaques en milieu hospitalier à Lomé (Togo)

**DOI:** 10.11604/pamj.2015.20.168.4979

**Published:** 2015-02-23

**Authors:** Abago Balaka, Toyi Tchamdja, Mohaman Awalou Djibril, Kodjo Agbéko Djagadou, Makilioubè Tchandana, Findibé Damorou, Aïssah Agbétra

**Affiliations:** 1 Service de Médecine Interne, CHU Sylvanus Olympio, Université de Lomé, Togo; 2 Ministère de la santé, Togo; 3 Service de Cardiologie, CHU Campus et Université de Lomé, Togo

**Keywords:** alvulopathies, épidémiologie, hôpital, Lomé, Togo, valvulopathy, epidemiology, hospital, Lomé, Togo

## Abstract

**Introduction:**

Notre étude a consisté en l'identification les principales valvulopathies retrouvées en milieu hospitalier à Lomé (Togo).

**Méthodes:**

Il s'agit d'une étude rétrospective, transversale, multicentrique menée du 1er janvier 2006 au 31 décembre 2010 et portant sur les dossiers de patients suivis dans le service de cardiologie du CHU Campus de Lomé.

**Résultats:**

Du 1^er^ janvier 2006 au 31 Décembre 2010,5412 patients ont été consulté dans le service de cardiologie du CHU Campus. Parmi eux, 241 (4,45%) présentaient une valvulopathie. On notait une prédominance féminine avec un sex-ratio H/F à 0,60. La moyenne d’âge était de 62,32 ans avec des extrêmes allant de 16 à 89 ans et un écart type de 14,27. Les antécédents le plus souvent retrouvés étaient l'hypertension artérielle (26,97%) et le diabète (8,29%). Parmi les motifs de consultations, les plus fréquents étaient la dyspnée (39,00%), les précordialgies (32,78%) et les palpitations (21,16%). A l'examen physique 30,70% des patients présentaient des signes de d'insuffisance cardiaque. A l’échographie, on notait des atteintes d'une seule valve (77,17%), de 02 valves (17,42%) ou 03 valves (5,4%). L'insuffisance mitrale (56,84%) et l'insuffisance aortique (30,70%) ont été les valvulopathies les plus fréquemment retrouvées. La maladie mitrale a été notée chez 05 patients. Les principales étiologies étaient dégénératives et ischémiques.

**Conclusion:**

Les valvulopathies sont relativement fréquentes à Lomé. L'insuffisance cardiaque est leur principal mode de révélation. Les plus retrouvées sont l'insuffisance mitrale et aortique.

## Introduction

Bien que les valvulopathies ne soient pas aussi fréquentes que les coronaropathies, l'insuffisance cardiaque ou l'hypertension artérielle, elles n'en représentent pas moins une entité clinique importante posant encore de nombreux problèmes de prise en charge [1]. Toutefois, au cours de ces dernières années, des progrès significatifs ont été réalisés dans la compréhension de la physiopathologie des valvulopathies. Les causes comme les caractéristiques cliniques des patients souffrant d'une atteinte valvulaire se sont modifiées [2, 3] et les méthodes d'exploration, qui reposaient naguère sur le cathétérisme, il y a quelques années, sont maintenant pratiquement remplacées par les méthodes non invasives, en particulier l’échocardiographie doppler cardiaque. Les résultats de la chirurgie se sont considérablement améliorés grâce à un diagnostic plus précoce, aux progrès de la réanimation postopératoire et à l'utilisation plus large de la chirurgie réparatrice, d'où l'importance du diagnostic précoce de ces valvulopathies dans nos milieux. Ainsi pour notre travail, nous nous sommes fixés comme objectifs: d’évaluer la prévalence des valvulopathies en milieu hospitalier, d'identifier les valvulopathies les plus fréquentes, et d'en recenser les principales causes.

## Méthodes

Il s'est agi d'une étude rétrospective, transversale, multicentrique qui a porté sur des patients ayant consulté ou ayant été hospitalisés au CHU Campus de Lomé au cours de la période du 1^er^ Janvier 2006 et le 31 Décembre 2010. Durant la période d’étude, 5412 patients ont été reçus en consultation et ou hospitalisés. Nous avons dans un premier temps sélectionné tous les dossiers des patients ayant une valvulopathie. Ensuite, nous avons réduit notre échantillon suivant les critères suivants: être âgé de quinze ans au moins, avoir fait une échocardiographie-doppler réalisé par un cardiologue, montrant au moins une valvulopathie (quel que soit son grade) avec épaississement, remaniement ou mutilation des feuillets valvulaires. N'ont pas été inclus, tous les patients ne répondant pas aux critères sus cités. Les données épidémiologiques, cliniques et para cliniques relatives à chaque patient hospitalisé ou ayant été reçu en consultation externe ont été consignées dans un formulaire que nous avons conçu pour la collecte des données.

## Résultats

### Caractéristiques générales de la population étudiée

Au total, 5412 patients ont consulté et ou hospitalisé dans le service de cardiologie pendant notre période d’étude, dont 241 présentaient une valvulopathie organique et/ou fonctionnelle. Soit une fréquence hospitalière de 4,45%. On notait une prédominance féminine avec un sex-ratio à 0,60. La moyenne d’âge était de 62,32 ans avec des extrêmes allant de 16 à 89 ans et un écart type de 14,27.

### Signes cliniques et facteurs de risques cardiovasculaires

Quatre-vingt-quatre patients (34,85%) avaient été déjà hospitalisés. Les motifs de consultations les plus fréquents étaient la dyspnée (39,00%), les précordialgies (32,78%) et les palpitations (21,16%). Des antécédents pathologiques ont été retrouvés dans la moitié des cas. Ainsi soixante-cinq patients (26,97%) avaient une HTA connue, 20 patients (8,29%) avaient un diabète. La tension artérielle, prise chez 189 patients était normale chez 54 d'entre eux (28,57%). On a noté une hyperthermie chez 12 patients soit 22,26%. Soixante-quatorze patients (30,70%) présentaient des signes d'insuffisance cardiaque

### Les valvulopathies cardiaques et signes associées

L’échographie cardiaque a permis de noter l'atteinte d'une seule valve chez 77,17% des patients, de 02 valves (17,42%) et de 03 valves (5,4%) comme présenté dans le tableau (Tableau 1). L'insuffisance mitrale (56,84%) et l'insuffisance aortique (30,70%) ont été les valvulopathies les plus retrouvées. La maladie mitrale a été retrouvée chez 05 patients (Tableau 2). La recherche étiologique a permis de noter la pathologie dégénérative (56,84%) et celle ischémique (11,2%) comme principales causes des valvulopathies chez nos patients (Tableau 3). Sur les 122 patients ayant bénéficié d'une radiographie thoracique, trente-cinq (28,68%) présentaient une cardiomégalie, 03 patients un déroulement de grosse aortique, tandis que 84 (60,8%) avaient une radiographie du thorax normal. Divers anomalies ont été retrouvées à l’électrocardiogramme chez nos patients: troubles de conduction (27,88%), signes d'ischémie (25,96%), extrasystoles (16,34%).

**Tableau 1:** Répartition selon les nombre de valves atteintes

	Effectifs	Pourcentage (%)
Atteinte Monovalvulaire	185	77,17
Atteinte Bivalvulaire	42	17,42
Atteinte Trivalvulaire	13	5,41

**Tableau 2:** Tableau de répartition des différentes valvulopathies

	Effectifs	Pourcentage (%)
Insuffisance mitrale	137	50,84
Insuffisance aortique	74	30,70
Rétrécissement aortique	65	26,97
Insuffisance triscuspidienne	18	07,46
Insuffisance pulmonaire	7	02,90
Rétrécissement mitrale	7	02,90

**Tableau 3:** Répartition de patients selon l’étiologue

	Effectifs	Pourcentage(%)
Pathologie dégénérative	137	56,84
Pathologie ischémique	27	11,20
RAA*	15	06,22
Autres	62	25,73
Total	241	100

* RAA: Rhumatisme articulaire aigüe

## Discussion

Dans notre étude, la fréquence des valvulopathies a été estimée à 4,45%. Ces résultats sont supérieurs à ceux retrouvés aux Etats-Unis [4] qui avait noté une fréquence de 2,5% dans la population générale. Cependant, elle est inférieure à celle de 9,4% retrouvée en milieu hospitalier au Congo [5] aucours de laquelle le point d'entrée des patients dans l’étude était l'insuffisance cardiaque. La prédominance féminine notée dans notre série n'avait pas été faite aux Etats-Unis [4] chez qui on ne notait pas d'ascendance d'un sexe sur l'autre. La prédominance féminine constatée dans notre étude serait due à la plus grande espérance de vie de la femme dans notre pays. Une grande proportion des patients de notre étude était des personnes âgées. Le vieillissement de la population avec l'augmentation de l'espérance de vie pourrait expliquer cette proportion importante des personnes âgées. La même remarque a été faite par Lung et al [6] lors une revue de la littérature.

Du point de vue clinique, 34.85% des patients présentant une valvulopathie avaient été hospitalisé pour insuffisance cardiaque et c'est lors du bilan qu'on a retrouvé la valvulopathie. Les motifs de consultation les plus fréquents étaient la dyspnée. (39%) les précordialgies (32,78%) et les palpitations (21,16%). En Madagasscar [7], les mêmes constats avaient été fait lors d'une étude sur les valvulopathies rhumatismales. Comme Lung et al [6], nos patients valvulopathes avaient comme facteurs de risques cardiovasculaires associées, l'HTA (27,9%), le diabète (08,29%), l'alcoolisme (07,46%). Ces auteurs avaient en effet remarqué que la présence de valvulopathies étaient associées à de nombreuses comorbidités dont les plus fréquentes étaient le diabète et l'HTA; qui peuvent de façon indirecte être les causes de ces valvulopathies. Par ailleurs, soixante-quatorze de nos patients présentaient les signes d'insuffisance cardiaque. Divers études américaines et africaines [8-10] avaient aussi noté les valvulopathies comme principales étiologies des insuffisances cardiaques. La défaillance cardiaque est selon Acar et al [11], la complication presque inéluctable de toute valvulopathie sévère. Au plan échocardiographique, on avait retrouvé dans 77,17% de cas une atteinte mono valvulaire. Cette fréquence élevée des atteintes mono valvulaires avait été aussi signalée lors de l'Euro Heart Survey [12] où les auteurs avaient noté un taux à 58%. De plus, les valvulopathies aortiques (57.67%) étaient plus fréquents que les valvulopathies mitrales dans notre série, ce qui est comparable aux constatations faites dans l'Euro Heart Survey [12] avec un taux à 56%. Par contre, dans l’étude faite par N'komo et al [4] dans la population Américaine, on notait une prédominance des valvulopathies mitrales.

L'insuffisance mitrale a été retrouvée chez 50,84% de nos patients; ce taux est supérieur à celui retrouvé dans l'Euro Heart Survey où les auteurs ont estimé un taux de 24,8%. Cette insuffisance mitrale souvent secondaire à un prolapsus valvulaire, ne s'accompagne d'une insuffisance mitrale sévère que dans 3,5% des cas [13]. L'insuffisance aortique a été notée chez 30,70% de nos patients. Lung B et al [6] à quant à lui retrouvé un taux plus bas à 10,4% de même que Singh et al [14]. Le rétrécissement aortique a été noté dans notre série chez 26,97% des patients. Un taux similaire à 33,9% a été retrouvé dans l'Euro Heart Survey. Le rétrécissement est ici probablement secondaire à une sclérose aortique dont la fréquence augmente avec l’âge [15]. En ce qui concerne le rétrécissement mitral, sa fréquence est estimé à 2,97% dans notre série. Dans l’étude Euro Heart Survey son taux était à 12%. Les taux bas retrouvés s'expliquent par le fait que le rétrécissement mitral est essentiellement dû au rhumatisme articulaire aigu (RAA), lequel est actuellement en régression dans tous les pays. Quant aux valvulopathies droites, leur taux était de 10.36%. Cette fréquence est nettement supérieure à celle rapportée par l'Euro Heart Survey chez qui il était noté 1.2% de valvulopathies droites.

Au plan étiologique, les causes dégénératives (56,84%) et ischémiques (11,20%) étaient les plus fréquentes. Lung et al [13] signalaient aussi dans leur étude une prédominance des causes dégénératives suivies des causes rhumatismales et ischémiques respectivement à 61,05%, 21,63% et 6,12%. Cette étiologie dégénérative prédominante serait due à la proportion très élevée des personnes âgées dans les différentes études faites par Lung [6]. En effet, des études faites en Inde et au Pakistan ont retrouvé des taux nettement inférieurs de valvulopathies rhumatismales à 5 pour mille [16]. Cependant ces résultats semblent être sous-estimés car des études récentes faites au Cambodge et au Mozambique montraient des taux de valvulopathies rhumatismales entre 21,5 et 30,4 pour 1000 après un dépistage échographique [17].

## Conclusion

Les valvulopathies sont relativement fréquentes au Togo. Les atteintes d'une seule valve étaient les plus fréquentes. Les insuffisances mitrale et aortique étaient les plus rencontrées. Leur principal mode de décompensation est l'insuffisance cardiaque Notre étude montre encore une fois l'intérêt du contrôle et de la prise en charge des facteurs de risque cardiovasculaires eu égard aux nouvelles donnes telles que le vieillissement progressif de notre population.
